# Use of contrast-enhanced ultrasound in pediatrics

**DOI:** 10.1590/0100-3984.2020.0167

**Published:** 2021

**Authors:** Marcelo Straus Takahashi, Mauricio Gustavo Ieri Yamanari, Lisa Suzuki, Ésio Fortaleza Nascimento Chaves Pedrosa, Roberto Iglesias Lopes, Maria Cristina Chammas

**Affiliations:** 1 Instituto de Radiologiado Hospital das Clínicas da Faculdade de Medicina da Universidade de São Paulo (InRad/HC-FMUSP), São Paulo, SP, Brazil.; 2 Instituto da Criança do Hospital das Clínicas da Faculdade de Medicina da Universidade de São Paulo (ICr/HC-FMUSP), São Paulo, SP, Brazil.; 3 Pediatric Urology Sector, Department of Urology, Hospital das Clínicas da Faculdade de Medicina da Universidade de São Paulo (HC-FMUSP), São Paulo, SP, Brazil.

**Keywords:** Pediatrics, Ultrasonography, Contrast media, Microbubbles, Pediatria, Ultrassonografia, Meio de contraste

## Abstract

Although contrast-enhanced ultrasound has been shown to provide considerable benefits, particularly in pediatric patients, it is still used relatively rarely in Brazil. It has proven to be a safe technique, and adverse effects are rare. In this review, we address the technique and main applications of contrast-enhanced ultrasound in the pediatric population, including the evaluation of focal liver lesions, abdominal trauma, kidney grafts, liver grafts, bowel loops, and vesicoureteral reflux. It is important for pediatric radiologists to be acquainted with this promising tool, understanding its applications and limitations.

## Abstract (pt)

A ultrassonografia com contraste de microbolhas é um método ainda pouco utilizado no Brasil, mas que tem grandes vantagens, ainda mais na faixa pediátrica, como ausência de radiação e dispensa de sedação/anestesia. O contraste de microbolhas tem se mostrado bastante seguro e com raríssimas reações adversas graves. Apresentaremos nesta revisão a técnica para o uso desse contraste na ultrassonografia, bem como as principais aplicações na faixa pediátrica, como avaliação das lesões hepáticas focais, do trauma abdominal, do enxerto hepático e renal nos transplantes, das alças intestinais e da pesquisa de refluxo vesicoureteral. É importante que radiologistas que exerçam funções em centros pediátricos conheçam essa ferramenta promissora.

## INTRODUCTION

Ultrasound is one of the main diagnostic imaging tools in pediatrics. It is a widely available noninvasive examination that does not require ionizing radiation or anesthesia, as well as having excellent anatomical resolution (especially in younger patients). Considered the examination of choice in many clinical scenarios, ultrasound is used almost universally in the field of pediatrics.

Ultrasound contrast media have been available on the market since the end of the 1990s^(1)^. Microbubbles are basically gas-liquid emulsions that generate an intense signal when exposed to ultrasound, which is why they are also known as “echo enhancers”. This new method has further broadened the range of ultrasound applications, allowing the microvasculature of organs to be evaluated, as well as intracavitary injection and the quantification of perfusion, to name a few examples^(2)^.

In pediatrics, the clinical application of contrast-enhanced ultrasound has lagged in relation to its use in adults, although recent advances in pediatric radiology research and the approval by regulatory agencies of certain specific uses have resulted in a progressive increase in the clinical use of this technique in pediatric patients^(3)^. Therefore, contrast-enhanced ultrasound in children has gained more and more prominence, especially in the evaluation of the liver, kidneys, and bowel loops, as well as in the diagnosis of vesicoureteral reflux, thus establishing itself as a viable alternative-and in some cases even a preferable alternative-to other diagnostic techniques.

## MICROBUBBLE CONTRAST AGENTS

The development of microbubble contrast agents dates back to the studies of Gramiak et al.^(4)^, in 1968, who noted what they called the “cloud of echoes” when they analyzed the effects of injecting saline solution through an intra-aortic catheter.

For a long time, the application of microbubble contrast agents was limited, especially because there was no product that was able to persist in the intravascular environment after passing through the pulmonary circulation. The first contrast agents that were more stable and were therefore able to reach the systemic circulation efficiently^(5)^, did not emerge until the second half of the 1990s.

Currently, the vast majority of commercially available microbubble contrast agents consist of an inert gas encapsulated in a shell. It should be noted that some authors classify contrast agents as “first generation” when the gas within the shell has a lower diffusion coefficient and is therefore less stable and as “second generation” when the gas within the shell has a higher diffusion coefficient and is therefore more stable. Currently, the commercially available contrast media are all second generation contrast agents.

The only contrast medium approved by the US Food and Drug Administration for use in pediatric patients is Lumason/SonoVue (Bracco Diagnostics Inc., Monroe Township, NJ, USA), which is indicated for the evaluation of focal liver lesions and for the investigation of vesicoureteral reflux. Any other contrast media and applications are considered off-label in most countries, including Brazil.

## SAFETY

There are currently two main routes of administration for contrast-enhanced ultrasound in children: intravenous application and intracavitary application.

Currently, the only absolute contraindication to the use of SonoVue (the only microbubble contrast agent commercially available in Brazil at this writing) is a history of hypersensitivity to some component of the contrast medium itself.

Intravenous application of microbubble contrast is recommended in the evaluation of the perfusion of various tissues and organs, such as the liver, kidneys, and bowel loops. After the intravenous administration of the contrast medium, the microbubbles remain restricted to the intravascular space, without entering the interstitium or parenchyma of the organs. Second-generation contrast agents usually persist in the circulation for approximately 10 min, and the gas is gradually excreted by the lungs as the microbubbles burst. The safety profile of intravenous application was extensively studied in a large cohort study of 23,188 adult patients who underwent these examinations, side effects being observed in only 29 cases, none of which evolved to death-severe in three, moderate in three, and mild in 23^(6)^. In a recent study of 312 children undergoing this type of examination, mild cutaneous reactions were observed in three patients and hypotension (reversed after pharmacological measures) was observed in three patients^(7,8)^.

In the vast majority of cases, the intracavitary application of microbubble contrast in pediatric patients is used in order to investigate vesicoureteral reflux. In this type of examination, also known as urosonography, the contrast medium is injected into the bladder by a bladder probe. Intracavitary application has proven to have an excellent safety profile, a retrospective study of 4,313 examinations in children showing that there were no adverse reactions that could be related exclusively to the use of contrast^(9)^.

## TECHNIQUE

A scanner equipped with specific contrast-enhanced ultrasound hardware and software is required in order to perform contrast-enhanced ultrasound. In general, it is desirable that the scanner be able to generate images based on inverted pulse harmonics, with pulse subtraction, to achieve greater contrast enhancement.

Because the interaction between the ultrasound beam and the microbubbles provokes more rapid destruction of the contrast agent, a low mechanical index (usually below 0.1) and the focus centered in the deeper portion of the image are needed in order to mitigate this destructive effect and increase the half-life of the contrast agent.

Prior to the injection of contrast medium, the regions of interest should always be analyzed with the ultrasound scanner in the contrast-specific mode in order to identify possible points that have hyperechoic areas, thus avoiding any uncertainties that may arise following the injection.

For examinations in which quantitative perfusion analysis is desired, a video clip is acquired with the transducer in a static position from prior to the injection until at least two minutes afterward, to perform a dynamic analysis of the region of interest.

## APPLICATIONS

### Focal liver lesions

In children, focal liver lesions may be detected as incidental findings during an abdominal evaluation for pain or other unrelated causes, in previously healthy children, or even in children with an underlying disease (liver disease or neoplasia).

The incidence of liver lesions in children is low (0.4-1.9 cases per 1,000,000 population) and is typically benign in approximately one third of the cases^(10)^.

Conventional ultrasound is the initial examination of choice for the evaluation of focal liver lesions in children and, when used in association with a color Doppler study, provides great assistance in narrowing the differential diagnosis on the basis of characteristics such as calcifications, vascularization, size/number of lesions, and vascular involvement^(11,12)^. However, in some cases, the findings are inconclusive and an additional examination is required, typically axial computed tomography or magnetic resonance imaging, which, despite several advantages, also present more risks and higher costs.

Recent studies have shown that contrast-enhanced ultrasound presents similar or even greater sensitivity and specificity than do axial methods for the diagnosis of focal liver lesions, often serving to elucidate the inconclusive results of computed tomography and magnetic resonance imaging^(13)^. In a cohort of 44 pediatric patients, Jacob et al.^(14)^ demonstrated that contrast-enhanced ultrasound had a specificity of 98% for reclassifying nodules categorized as indeterminate in other types of examinations as benign nodules.

The assessment of focal liver lesions by contrast-enhanced ultrasound in children is always preceded by a thorough ultrasound evaluation without liver contrast, in order to identify and characterize the specific lesion to be evaluated.

Contrast-enhanced ultrasound begins once the lesion is localized. The dose approved by SonoVue for hepatic evaluation, which is approved by the Food and Drug Administration for children, is 0.03 mL/kg, with a maximum dose of 2.4 mL. The first two minutes of the examination should be recorded on the scanner as a video clip. Static images should be obtained up to five minutes from the start of injection for further analysis. If the results of the examination are not satisfactory or if there is more than one suspected lesion, an additional dose of the contrast agent equal to that of the initial injection can be considered.

**Figure 1:**
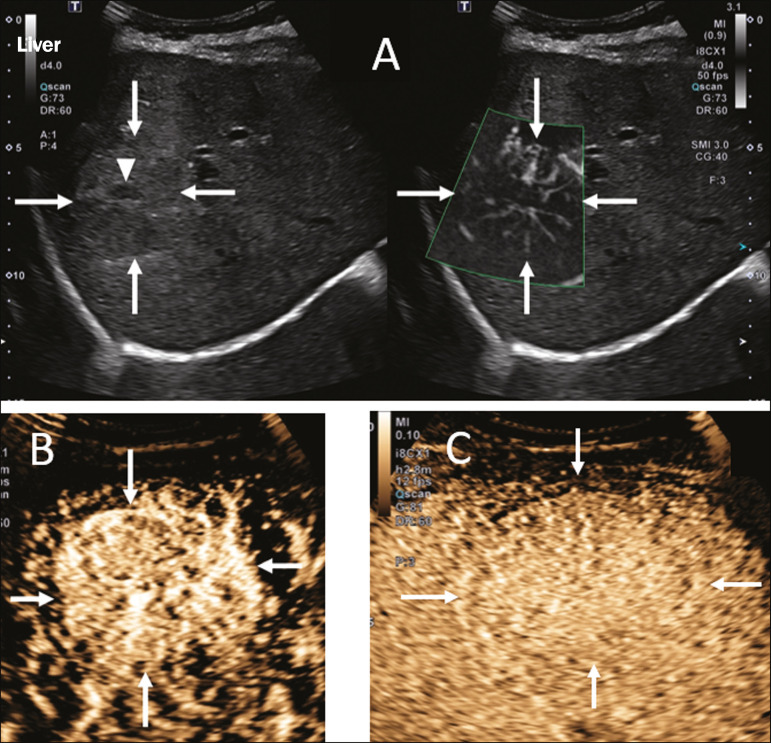
A 14-year-old patient with a liver nodule identified as an incidental finding on ultrasound. **A:** Simultaneous B-mode and Doppler intensity ultrasound images (left and right, respectively), showing a nodule (arrows on the left) that is slightly hypoechoic in relation to the liver parenchyma, with a hypoechoic center (central scar; arrowhead on the left). On the color Doppler image, a central artery appears as a star-shaped image (arrows on the right). **B:** After administration of the contrast medium, ultrasound showed a central artery in a star shape (arrows). **C:** Subsequent centrifugal enhancement, with complete contrast filling and persistence in the delayed phases (arrows).

**Figure 2:**
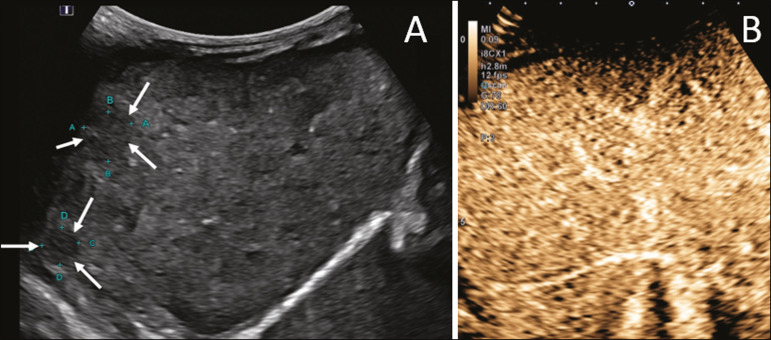
A 12-year-old patent with chronic liver disease and multiple nodules on ultrasound. **A:** B-mode ultrasound showing multiple hypoechoic nodules (arrows). **B:** On contrast-enhanced ultrasound, note that the enhancement of the nodules is similar to that of the adjacent liver parenchyma, on the arterial, portal venous, and delayed-phase images, without washout, consistent with regenerative nodules.

**Figure 3:**
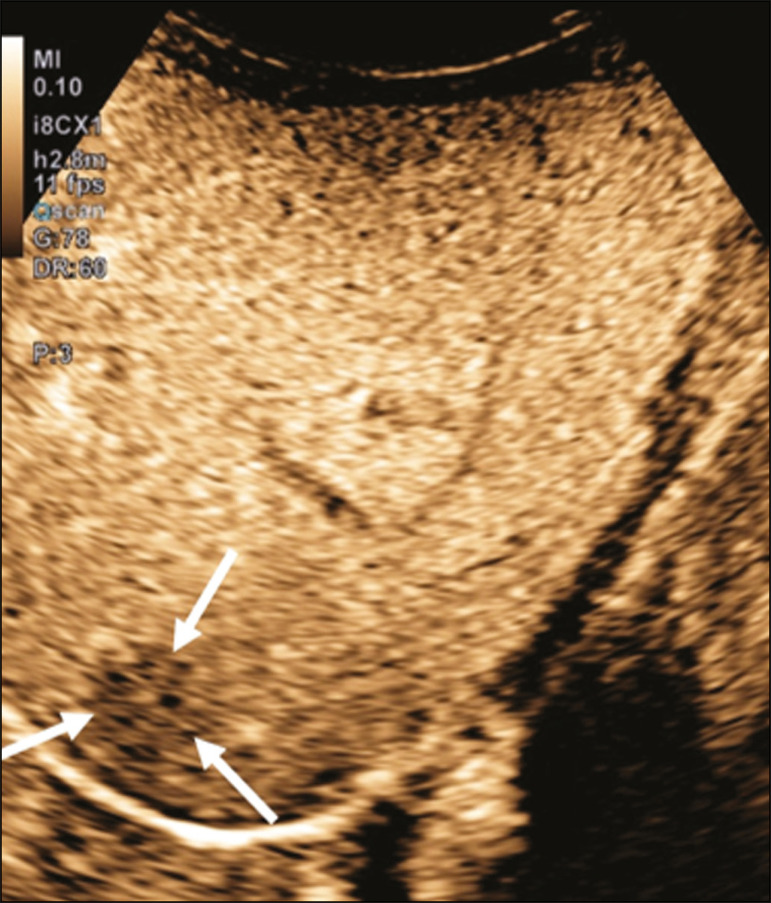
A seven-year-old patient with a history of resected right adrenal carcinoma. A follow-up conventional ultrasound scan had detected hypoechoic and heterogeneous nodules. Contrast-enhanced ultrasound showing lesions with early washout in relation to the liver parenchyma (arrows), consistent with metastasis.

The enhancement patterns of focal liver lesions in contrast-enhanced ultrasound have been well documented^(13)^. Benign nodules typically have specific enhancement patterns, depending on their nature (Figures 1 and 2), whereas malign nodules typically show a more consistent pattern of intense enhancement in the arterial (hypervascular) phase and early washout during the portal venous phase (Figure 3).

### Abdominal trauma

**Figure 4:**
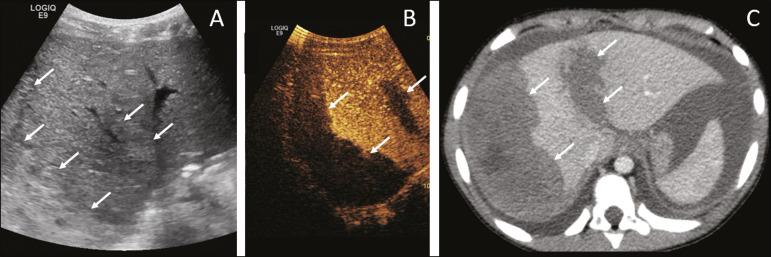
A nine-year-old patient with abdominal trauma from a domestic accident. **A:** Conventional ultrasound showing slightly hypoechoic and heterogeneous areas in the right lobe of the liver, with ill-defined borders (arrows). **B:** After administration of contrast medium, ultrasound clearly showed a laceration in the right lobe (arrows). **C:** Computed tomography scan acquired on the same day, showing lacerations (arrows).

The use of focused assessment with sonography for trauma is currently one of the pillars of care for patients with abdominal trauma. This technique provides great sensitivity in the detection of free fluid in the abdominal cavity. This finding is usually related to the lesion of an abdominal organ. Unfortunately, the technique is limited to more specific identification of the lesion site, because lacerations of solid viscera are hardly ever characterized directly on ultrasound imaging. The use of intravenous contrast medium overcomes this limitation because it allows the areas of visceral laceration to be determined^(15)^, as illustrated in Figure 4.

In the context of abdominal trauma, contrast-enhanced ultrasound can be used basically in two scenarios: the initial evaluation of patients who are hemodynamically stable; and the follow-up of previously diagnosed traumatic lesions.

In the initial evaluation of trauma, administration of the contrast medium is divided into two boluses: the first is used to evaluate the organs present in the right hypochondrium (liver, right kidney, right adrenal gland, and part of the pancreas) and the second dose to evaluate the organs present in the left hypochondrium (spleen, left kidney, left adrenal gland, and part of the pancreas). The evaluations should always begin with the kidney, because contrast enhancement occurs faster in the kidney than in the liver or the spleen.

In the follow-up examination of traumatic injuries, there is no need to use two boluses, because the lesions have been previously diagnosed and their location is known. Visceral lacerations have a very specific imaging pattern and appear as areas without enhancement after injection of the contrast medium (Figure 4).

### Transplantation in children

The two main applications of contrast-enhanced ultrasound in the evaluation of solid organ transplantation in children are kidney transplantation^(16)^ and liver transplantation^(17)^. The great advantage of this method is that it provides a more detailed evaluation of vascularization and of graft perfusion, precluding the need to move the patient to the diagnostic center.

**Figure 5:**
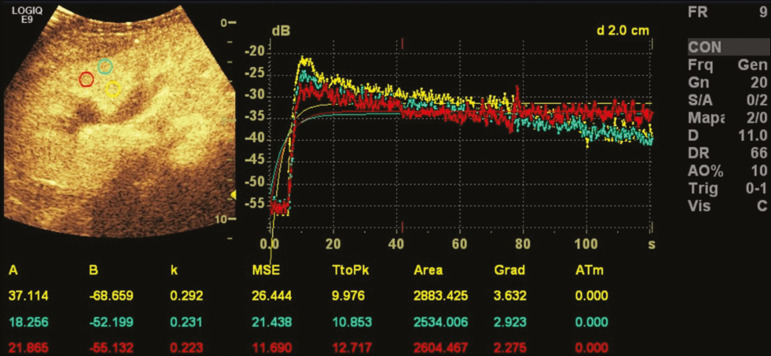
Quantitative assessment of perfusion without signs of graft dysfunction in a patient who underwent kidney transplantation. Note the homogeneous contrast enhancement of the graft, the enhancement curves showing a preserved time to peak, without perfusion asymmetry between the renal cortex and the renal pyramids.

In cases of renal transplantation, contrast-enhanced ultrasound can be used for the qualitative evaluation of graft perfusion, indicating areas of infarction that do not show enhancement in the contrast-specific mode, as well as for the quantitative evaluation of renal parenchyma perfusion (Figure 5). In the evaluation of quantitative perfusion, the transducer is held in a static position prior to and during the injection of the contrast medium in order to generate a video at least two minutes in length. The video is processed after the injection and, intensity curves are generated for the determined regions of interest, which can help differentiate, in particular, cases of acute tubular necrosis rejection.

In cases of liver transplantation, the main application of contrast-enhanced ultrasound is the evaluation of hepatic arterial patency in the initial postoperative period. Hepatic artery thrombosis is one of the main causes of graft loss, and it is not uncommon for patients to undergo computed tomography angiography or conventional angiography to confirm the diagnosis after an inconclusive result of a Doppler study of a graft. Contrast-enhanced ultrasound is useful as an intermediate examination, with greater accuracy than color Doppler ultrasound and the advantage of being able to be performed at the bedside, as well as having few contraindications.

### Bowel loops

**Figure 6:**
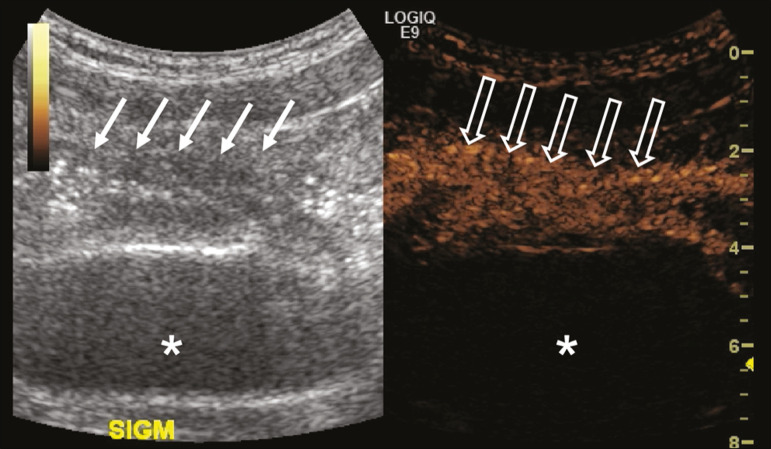
A 15-year-old patient, under follow-up treatment for Crohn’s disease, with abdominal pain and suspected exacerbation of the disease. Images after intravenous injection of microbubble contrast medium, showing wall thickening of the sigmoid loop (arrows) on B-mode ultrasound; in the contrast-specific mode, note the intense microbubble contrast parietal enhancement of the loop (open arrows). The anechoic structure at the bottom of the image is the full bladder (asterisks).

The main indication for the use of contrast-enhanced ultrasound of the gastrointestinal tract is the evaluation of patients with inflammatory bowel disease (Figure 6). It can be used in order to estimate the inflammatory activity in the loop, to distinguish between fibrosis and inflammation in a stenosed segment, and to monitor the effect of treatment of the disease^(18)^.

Contrast-enhanced ultrasound allows objective data to be obtained (peak intensity in decibels, time to peak, area under the curve, and contrast washout time) from a time-intensity curve.^(19)^ To acquire that curve, the radiologist first needs to determine, on conventional ultrasound, which intestinal segment is the region of interest (typically the segment most affected by the disease). After the region of interest has been selected, the transducer is maintained in a static position, scanning the same region during and for at least two minutes after injection of contrast medium. In addition, the intensity and pattern of the enhancement of the bowel loop and the adjacent mesentery are evaluated subjectively. Contrast-enhanced ultrasound even evaluates the complications of inflammatory bowel disease, such as abscesses and fistulae. In the abscess, the center is not enhanced by the microbubble contrast agent. To evaluate the fistulae, the contrast agent can be injected through the orifice in order to evaluate the fistulous tract.

Although contrast-enhanced ultrasound is not the method of choice for the evaluation of tumors and intestinal polyps, it can be useful in quantifying the vascularization of lesions supposedly located in the intestinal mucosa and can guide biopsies to avoid necrotic regions. It can also be useful in cases of multiple-organ transplantation, by evaluating perfusion of the intestinal wall in the same manner as that used for the detection of intestinal ischemia.

Although its use is more restricted in cases of other inflammatory diseases such as appendicitis, diverticulitis, and appendagitis, when the diagnosis of abscess is uncertain, the peripheral enhancement and the absence of internal contrast enhancement can be highly useful^(20)^.

### Voiding urosonography

One of the main applications of contrast-enhanced ultrasound is voiding urosonography. In this application, the contrast medium is injected into the bladder with a probe and is most often used for the investigation of vesicoureteral reflux^(21)^.

Voiding urosonography is the main alternative to voiding cystourethrography (VCUG) in children, excellent agreement between the two techniques having been demonstrated. In a recent review of the literature, Chua et al.^(22)^ observed that 90% sensitivity and 93% specificity in comparison with VCUG. In addition, voiding urosonography presents excellent spatial and temporal resolution and has an excellent safety profile.

With the patient positioned on the examination table, the first step is to perform conventional ultrasound of the kidneys and urinary tract, for structural assessment. At the end of the conventional examination, the urinary tract is examined and documented in contrast-specific mode prior to contrast administration, in order to identify possible areas of high echogenicity that may generate uncertainty in the contrast phase. The patient is then fitted with a catheter, which is used in order to empty the bladder. After being emptied, the bladder is slowly filled with diluted contrast medium (typically diluted to 2% in saline solution) in a manner similar to that used for VCUG. During bladder filling and voiding (the patient urinates around the catheter, which usually occupies one-third of the diameter of the urethra), the examiner constantly evaluates the paths of the ureters and the renal pelvis in contrast-specific mode to determine whether there is vesicoureteral reflux. This filling/emptying process is performed at least twice.

**Figure 7:**
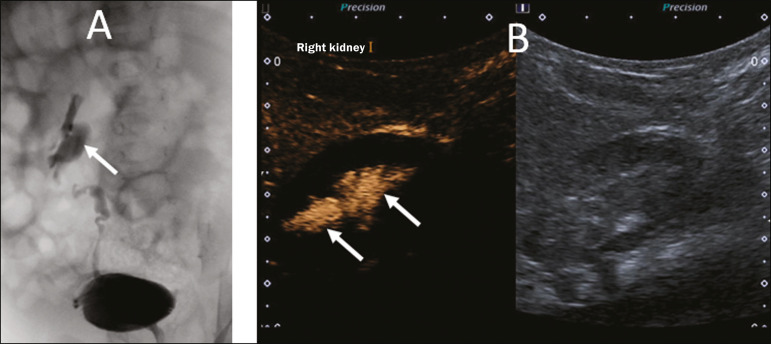
A three-month-old patient undergoing investigation of vesicoureteral reflux. A: Voiding cystourethrography showing vesicoureteral reflux with filling of the renal pelvis and calyces by contrast medium (arrow). B: Voiding urosonography in the contrast-specific mode, showing reflux of microbubble contrast medium (injected through the bladder catheter) to the renal pelvis (arrows).

The diagnosis of vesicoureteral reflux is established when microbubbles are identified in the ureter or renal pelvis (Figure 7), and the reflux can be graded in a manner similar to that employed in the international system of radiographic grading of vesicoureteric reflux^(23)^.

The main limitation of voiding urosonography in relation to VCUG appears in patients in whom ultrasound access to the kidneys is limited (severe scoliosis) or in patients in whom there is reflux to the ureters only, because the visualization on ultrasound can be more difficult in such patients. It is of note that the treatment of vesicoureteral reflux, either by antibiotic prophylaxis or by surgery, is recommended for most patients who are diagnosed with high-grade reflux.

## CONCLUSION

The application of contrast-enhanced ultrasound in children has increased significantly in recent years and should increase even more, given that government entities worldwide have recently approved it for use in urological and liver applications. It is important for radiologists, especially those who work at pediatric centers, to be aware of this tool, its scope, and its limitations. Contrast-enhanced ultrasound has proven to be a safe, effective technique in many clinical scenarios. It can reduce the risks associated with sedation, ionizing radiation, and the possible reactions associated with other contrast media.

## References

[1] Paefgen V, Doleschel D, Kiessling F (2015). Evolution of contrast agents for ultrasound imaging and ultrasound-mediated drug delivery. Front Pharmacol.

[2] Piscaglia F, Nolsøe C, Dietrich CF (2012). The EFSUMB guidelines and recommendations on the clinical practice of contrast enhanced ultrasound (CEUS): update 2011 on non-hepatic applications. Ultraschall Med.

[3] Ntoulia A, Anupindi SA, Darge K (2018). Applications of contrast-enhanced ultrasound in the pediatric abdomen. Abdom Radiol (NY).

[4] Gramiak R, Shah PM (1968). Echocardiography of the aortic root. Invest Radiol.

[5] Ignee A, Atkinson NSS, Schuessler G (2016). Ultrasound contrast agents. Endosc Ultrasound.

[6] Piscaglia F, Bolondi L, Italian Society for Ultrasound in Medicine and Biology Study Group on Ultrasound Contrast Agents (2006). The safety of Sonovue in abdominal applications: retrospective analysis of 23188 investigations. Ultrasound Med Biol.

[7] Mao M, Xia B, Chen W (2019). The safety and effectiveness of intravenous contrast-enhanced sonography in Chinese children - a single center and prospective study in China. Front Pharmacol.

[8] Rosado E, Riccabona M (2016). Off-label use of ultrasound contrast agents for intravenous applications in children: analysis of the existing literature. J Ultrasound Med.

[9] Riccabona M (2012). Application of a second-generation US contrast agent in infants and children-a European questionnaire-based survey. Pediatr Radiol.

[10] Chiorean L, Cui XW, Tannapfel A (2015). Benign liver tumors in pediatric patients - review with emphasis on imaging features. World J Gastroenterol.

[11] Chung EM, Cube R, Lewis RB (2010). From the archives of the AFIP: Pediatric liver masses: radiologic-pathologic correlation part 1. Benign tumors. Radiographics.

[12] Chung EM, Lattin Jr GE, Cube R (2011). From the archives of the AFIP: Pediatric liver masses: radiologic-pathologic correlation part 2. Malignant tumors. Radiographics.

[13] Anupindi SA, Biko DM, Ntoulia A (2017). Contrast-enhanced US assessment of focal liver lesions in children. Radiographics.

[14] Jacob J, Deganello A, Sellars ME (2013). Contrast enhanced ultrasound (CEUS) characterization of grey-scale sonographic indeterminate focal liver lesions in pediatric practice. Ultraschall Med.

[15] Armstrong LB, Mooney DP, Paltiel H (2018). Contrast enhanced ultrasound for the evaluation of blunt pediatric abdominal trauma. J Pediatr Surg.

[16] Álvarez Rodríguez S, Hevia Palacios V, Sanz Mayayo E (2017). The usefulness of contrast-enhanced ultrasound in the assessment of early kidney transplant function and complications. Diagnostics (Basel).

[17] Torres A, Koskinen SK, Gjertsen H (2019). Contrast-enhanced ultrasound for identifying circulatory complications after liver transplants in children. Pediatr Transplant.

[18] De Franco A, Marzo M, Felice C (2012). Ileal Crohn's disease: CEUS determination of activity. Abdom Imaging.

[19] Medellin A, Merrill C, Wilson SR (2018). Role of contrast-enhanced ultrasound in evaluation of the bowel. Abdom Radiol (NY).

[20] Ripollés T, Martínez-Pérez MJ, Paredes JM (2013). Contrast-enhanced ultrasound in the differentiation between phlegmon and abscess in Crohn's disease and other abdominal conditions. Eur J Radiol.

[21] Duran C, Beltrán VP, González A (2017). Contrast-enhanced voiding urosonography for vesicoureteral reflux diagnosis in children. Radiographics.

[22] Chua ME, Kim JK, Mendoza JS (2019). The evaluation of vesicoureteral reflux among children using contrast-enhanced ultrasound: a literature review. J Pediatr Urol.

[23] Lebowitz RL, Olbing H, Parkkulainen KV (1985). International system of radiographic grading of vesicoureteric reflux: International reflux study in children. Pediatr Radiol.

